# Novel circular RNA expression profiles reflect progression of patients with hypopharyngeal squamous cell carcinoma

**DOI:** 10.18632/oncotarget.17488

**Published:** 2017-04-27

**Authors:** Shengda Cao, Dongmin Wei, Xu Li, Jieyu Zhou, Wenming Li, Ye Qian, Zhanwang Wang, Guojun Li, Xinliang Pan, Dapeng Lei

**Affiliations:** ^1^ Department of Otorhinolaryngology, Qilu Hospital, Shandong University, Key Laboratory of Otolaryngology, NHFPC (Shandong University), Jinan 250012, P. R. China; ^2^ Department of Otorhinolaryngology, Shanghai Ninth People's Hospital, Shanghai Jiaotong University, School of Medicine, Shanghai 200011, P. R. China; ^3^ Department of Head and Neck Surgery, Houston, TX 77030, USA; ^4^ Epidemiology, The University of Texas MD Anderson Cancer Center, Houston, TX 77030, USA

**Keywords:** circRNAs, hypopharyngeal squamous cell carcinoma, microarray analysis, miRNA sponges, bioinformatics analysis

## Abstract

Circular RNAs (circRNAs), a novel class of endogenous noncoding RNAs, have been shown to have important roles in a number of diseases, including several types of cancers. We hypothesized that circRNAs are involved in the pathogenesis of hypopharyngeal squamous cell carcinoma (HSCC). To test our hypothesis, we initially compared the expression profiles of circRNAs in 4 paired HSCC and adjacent normal tissue samples by using a circRNA microarray. The microarray data showed that 2392 circRNAs, including 1304 upregulated and 1088 downregulated circRNA transcripts, were significantly dysregulated in the HSCC tissues. The 10 most dysregulated circRNAs from the microarray analysis were further validated in another 32 pairs of specimens using quantitative real-time polymerase chain reaction assays. These circRNAs might sponge microRNAs (miRNAs) in predicted circRNA-miRNA-mRNA networks. Bioinformatics analysis was also performed to predict possible pathways in which these networks might be involved. Finally, we analyzed the interaction between validated circRNAs and their potential cancer-related miRNA targets. We are the first to comprehensively delineate the expression profiles of circRNAs in HSCC and to provide potential candidates for future mechanism studies. Our study is potentially of critical significance in uncovering the roles of circRNAs in HSCC.

## INTRODUCTION

Hypopharyngeal squamous cell carcinoma (HSCC) is characterized by poor differentiation and early metastasis and is one of the most aggressive head and neck squamous cell carcinomas. Patients with HSCC are typically diagnosed with advanced-stage disease that is associated with a poor prognosis [1]. Despite improvements in comprehensive treatment strategies in recent years (including surgery, radiation therapy, and chemotherapy), patients with HSCC are still vulnerable to disease relapse, and the 5-year survival rate ranges from 25% to 40% [2]. Therefore, the identification of new prognostic markers and therapeutic targets is critical for improving the outcomes of patients with HSCC.

A new type of endogenous noncoding RNA, circular RNA (circRNA), is joining microRNA (miRNA) and long noncoding RNA (lncRNA) as an area of great interest in the field of RNA research [3]. Previously, circRNAs were misinterpreted as byproducts of splicing errors [4], but with the development of high-throughput sequencing technology and bioinformatics, circRNAs have been recognized as a class of widespread, abundant, stable, conserved, and tissue-specific endogenous noncoding RNAs in mammalian cells [5–7]. Produced by back-splice events, circRNAs are characterized by covalently closed loop structures with neither 5′ or 3′ polarities nor polyadenylated tails [8], rendering them resistant to degradation by RNase R or RNA exonuclease [9]. Recently, circRNAs have been found to sequester miRNAs [10–12] and regulate alternative splicing or transcription processes [13] as well as parental gene expression [7]. Even though the functions of most circRNAs still remain unclear [13], accumulated evidence indicates that circRNAs are probably involved in tumorigenesis and cancer progression. In addition to functioning as a miRNA sponge in mechanisms of circRNAs, other still unknown function of circRNAs, such as regulating alternative splicing, transcription or the expression of parental gene may also contribute to the progression of HSCC. A growing number of circRNAs are aberrantly expressed in colorectal cancer [14], hepatocellular carcinoma [15, 16], gastric cancer [17], and laryngeal squamous cell carcinoma [18] and have the potential to become new diagnostic or prognostic biomarkers. More importantly, the molecular mechanisms of some circRNAs and their clinical significance in cancers have been investigated [11, 12, 19].

Considering the emerging roles of circRNAs in cancers, we hypothesized that circRNAs might be involved in the tumorigenesis or progression of HSCC. To test our hypothesis, we first evaluated the circRNA expression profiles in HSCC and paired adjacent normal tissue samples (*n* = 4) by using microarray analysis. The circRNAs that were dysregulated in the HSCC tissues were then validated and confirmed by quantitative real-time polymerase chain reaction (qRT-PCR). A step-wise bioinformatics analysis was performed to predict pathways in which validated circRNAs might be involved.

## RESULTS

### CircRNA expression profiles in HSCC and adjacent normal tissues

**Figure 1:**
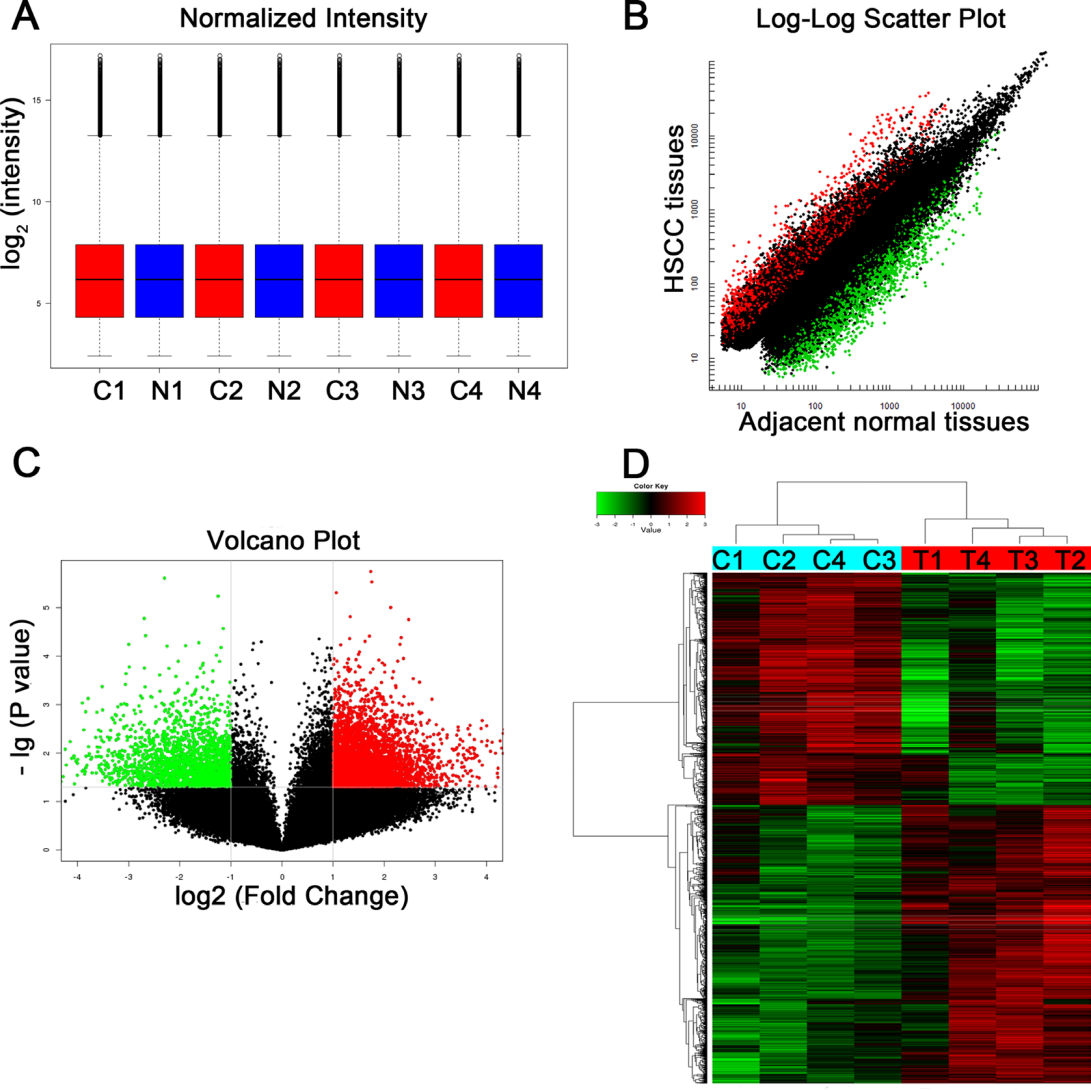
Different expression profiles of circRNAs in HSCC tissues versus adjacent normal tissues (**A**) A box plot for visualizing the distributions of normalized intensities in samples. (**B**) A scatter plot for revealing the difference in circRNA expression between HSCC tissue and adjacent normal tissues. (Red points stand for upregulated circRNAs with FC ≥ 2.0 in HSCC tissues and green points represent downregulated circRNAs) (**C**) A volcano plot showing significantly dysregulated circRNAs in HSCC tissues. (Red or green points represent upregulated or downregulated circRNAs in HSCC tissues, respectively (FC ≥ 2.0, *P* value < 0.05), respectively.) (**D**) Heat map and hierarchical clustering analysis revealed different circRNA expression profiles between HSCC tissues and adjacent normal tissues. Hierarchical clustering analysis for 8 samples in the tumor group (C1, C2, C3, and C4) and non-tumor group (T1, T2, T3, and T4).

A total of 87,935 circRNA targets were detected in the four paired HSCC tumor and adjacent normal tissues. A box plot showed that the distributions of the normalized intensities from all the datasets in the tested samples were essentially identical (Figure 1A). A scatter plot was used for visualizing the difference in circRNA expression in HSCC tumor tissues and paired normal tissues (Figure 1B). The red and green points represent upregulated and downregulated circRNAs, respectively, with fold change ≥ 2.0 (FC, the ratio of averaged signal values between two conditions) in HSCC tissues. Further, volcano plot filtering identified significantly differentially expressed circRNAs (FC ≥ 2.0, *P* value < 0.05) between the two groups of samples (Figure 1C). Consequently, 2392 circRNAs dysregulated in tumor tissues with FC ≥ 2.0 and *P* value < 0.05 were identified. Among them, 1304 were upregulated and 1088 were downregulated in tumor tissues as compared with paired adjacent normal tissues. Additionally, the top 10 upregulated and downregulated circRNAs sorted by their FC values (absolute value) were summarized in Table 1, along with other detailed information on FC values, *P* values, gene symbols, numbers of predicted targeted miRNAs from miRanda and TargetScan, etc.

**Table 1:** Selected top 10 altered expression of circRNAs in HSCC tissues by fold change (FC)

Top 10 upregulated circRNAs						
CircRNA ID	FC	*P* value	Chromosome	Strand	Gene symbol	No. miRNA targets
hsa_circ_0024109	40.295	0.009	11	−	MMP1	12
hsa_circ_0058143	30.341	0.022	2	−	FN1	3
hsa_circ_0058104	27.735	0.003	2	−	FN1	6
hsa_circ_0058106	26.222	0.014	2	−	FN1	2
hsa_circ_0058121	25.540	0.018	2	−	FN1	4
hsa_circ_0058107	23.066	0.002	2	−	FN1	11
hsa_circ_0024108	22.409	0.007	11	−	MMP1	10
hsa_circ_0058095	22.185	0.004	2	−	FN1	21
hsa_circ_0058115	20.418	0.003	2	−	FN1	28
hsa_circ_0058097	20.418	0.004	2	−	FN1	159
Top 10 downregulated circRNAs						
hsa_circ_0003441	23.548	0.007	13	+	TDRD3	0
hsa_circ_0001290	21.410	0.027	3	−	SETD2	0
hsa_circ_0050108	21.133	0.003	19	+	SSBP4	47
hsa_circ_0088635	20.274	0.005	9	+	GARNL3	0
hsa_circ_0002260	20.023	0.039	5	+	PAPD4	2
hsa_circ_0007646	19.457	0.031	4	+	DCUN1D4	0
hsa_circ_0082212	18.660	0.019	7	+	FLNC	73
hsa_circ_0001189	17.691	0.006	21	+	MORC3	3
hsa_circ_0036722	17.339	0.013	15	−	RHCG	24
hsa_circ_0087964	16.798	0.011	9	−	LPAR1	885

CircRNA ID was based on circBase (http://www.circbase.org/).

Hierarchical clustering analysis clearly showed differential circRNA expression between the tumor group and non-tumor group (Figure 1D), indicating the circRNA expression profiles in tumor tissues were different from those in adjacent normal tissues.

### Aberrant expression of circRNAs in chromosomes

**Figure 2:**
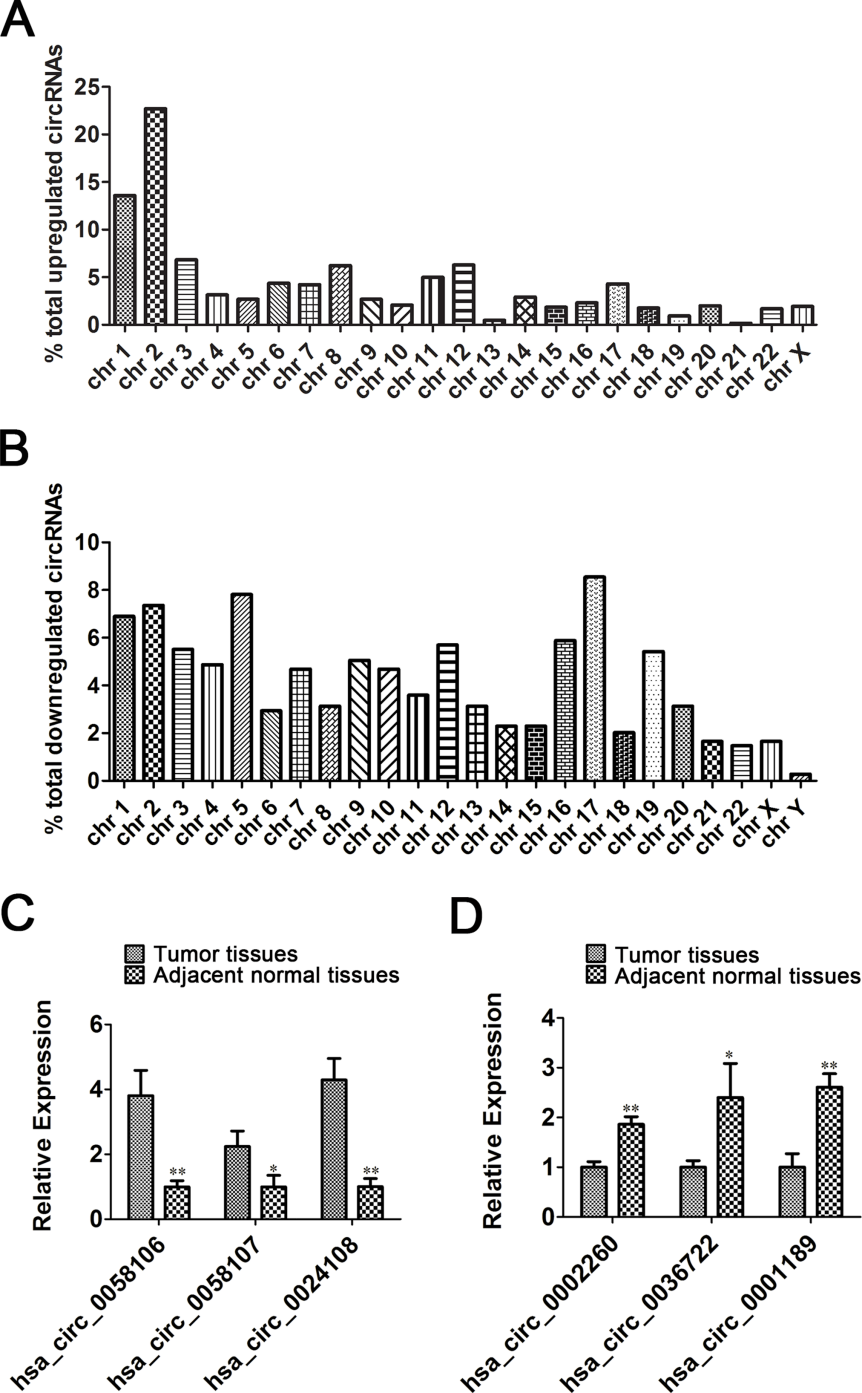
The distribution of significantly dysregulated circRNAs [(**A**) Upregulated and (**B**) downregulated circRNAs] and Validation of top 10 upregulated and downregulated circRNAs by qRT-PCR [(**C**) qRT-PCR assays verified 3 upregulated and (**D**) 3 downregulated circRNAs. The relative expression of each circRNA was normalized to its mean expression value in adjacent normal tissues, respectively. Data presented as mean ± SE. *P < 0.05, **P < 0.01].

We further categorized these significantly aberrantly expressed circRNAs according to the chromosomes they were transcribed from. The results revealed that these circRNAs were widely distributed among all chromosomes, including sex chromosomes. Among the upregulated circRNAs in tumor tissues, approximately 22.7% came from chromosome 2 (chr 2) and 13.6% from chr1, whereas the percentages of upregulated circRNAs from any other chromosome were less than 7% (Figure 2A). In contrast, the distribution of downregulated circRNAs was more balanced. The top five chromosomes with downregulated circRNAs were chr 17 (8.6%), chr 5 (7.8%), chr 2 (7.4%), chr1 (6.9%), and chr 16 (5.8%) (Figure 2B).

### Validation of identified circRNAs by qRT-PCR

We selected the top 10 upregulated and downregulated circRNAs based on the FC (fold change) values and significance (*P* values) from microarray data for validation. Divergent primers for those circRNAs were designed, and the appearance of one single peak in the melting curve confirmed the specificity of the amplified products for each circRNA (Supplementary Figure 1).

The expression levels of the 20 circRNAs were determined in another 32 pairs of HSCC tumor tissues and adjacent normal tissues using qRT-PCR. We found that the expression levels of hsa_circ_0058106, hsa_circ_0058107, and hsa_circ_0024108 were significantly higher in tumor tissues than in corresponding normal tissues (Figure 2C, *P < 0.05, **P < 0.01). Meanwhile, among the top 10 downregulated circRNAs, hsa_circ_0036722, hsa_circ_0002260, and hsa_circ_0001189 expression levels were significantly decreased in tumor tissues compared with normal controls (Figure 2D, *P < 0.05, **P < 0.01). These data were consistent with the results of the microarray data, indicating the reliability of these results. In addition, the six validated circRNAs might be promising candidates for future studies of molecular mechanisms.

### Prediction of miRNAs targeted by validated circRNAs

Increasing evidence indicates that circRNAs can sequester relevant miRNAs with miRNA response elements (MREs) to post-transcriptionally regulate gene expression [10]. Therefore, we predicted potential miRNA targets of the validated circRNAs using miRNA target predication software. The predicted miRNA targets of validated circRNAs are summarized in Table 2.

**Table 2:** Potential miRNA targets of validated circRNAs

CircRNA ID	Regulation	Gene symbol	Potential miRNA targets (No. MREs)
hsa_circ_0058106	Up	FN1	miR-185-3p (2); miR-4638-3p (2)
hsa_circ_0058107	Up	FN1	miR-185-3p (2); miR-3137 (2); miR-365b-5p (2); miR-4638-3p (2); miR-4673 (2); miR-4722-5p (2); miR-4726-3p (2); miR-6751-5p (2); miR-6752-5p (2); miR-6803-5p (3); miR-6815-5p (2)
hsa_circ_0024108	Up	MMP1	miR-185-3p (2); miR-296-3p (2); miR-4420 (2); miR-4646-3p (2); miR-4743-3p (2); miR-4755-5p (2); miR-623 (2); miR-670-5p (2); miR-6804-5p (2); miR-7108-5p (2)
hsa_circ_0036722	Down	RHCG	miR-1250-5p (2); miR-1254 (2); miR-1301-3p (2); miR-145-5p (2); miR-185-3p (2); miR-3127-5p (2); miR-3186-3p (2); miR-323b-5p (2); miR-3675-5p (2); miR-4435 (2); miR-4474-3p (2); miR-4715-3p (2); miR-4726-5p (2); miR-5088-3p (2); miR-6089 (3); miR-671-5p (2); miR-6757-5p(2); miR-6762-5p(2); miR-6767-5p(2); miR-6799-3p (2); miR-6836-5p (2); miR-6880-5p (2); miR-6895-5p (2); miR-8077 (2)
hsa_circ_0002260	Down	PAPD4	miR-1229-5p (2); miR-1301-3p (2)
hsa_circ_0001189	Down	MORC3	miR-3127-3p (2); miR-5088-3p (2); miR-6756-3p (2)

MREs: miRNA response elements

### Bioinformatics analysis of validated circRNA-miRNA-mRNA networks

**Figure 3:**
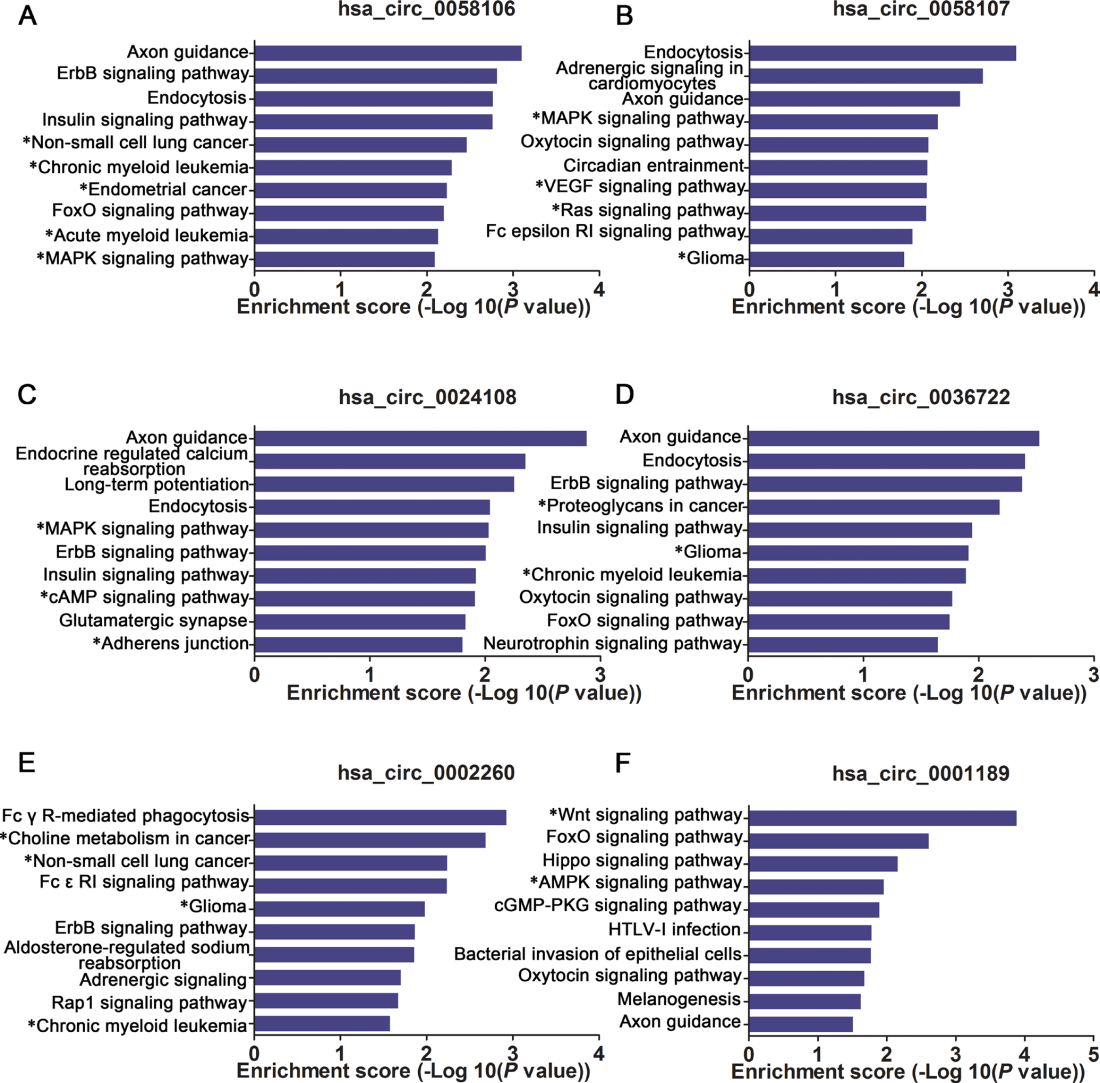
Bioinformatics analysis of (**A**) hsa_circ_0058106/ (**B**) hsa_circ_0058107/ (**C**) has_circ_00024108/ (**D**) has_circ_0036722/ (**E**) has_circ_0002260/ (**F**) has_circ_0001189 targeted circRNA-miRNA-mRNA networks. Target mRNAs of the 6 networks are functionally annotated by KOBAS and KEGG pathway analysis. Top 10 significant enriched pathway terms of the 6 networks.

We further established circRNA-miRNA-mRNA networks with validated circRNAs, their predicated miRNA targets, and downstream regulated mRNAs (data not shown). KEGG pathway analysis was performed to functionally annotate the predicted mRNA targets within the above networks. As shown in Figure 3, each of these networks has several significantly enriched cancer-related pathways (marked with asterisks). This information might help us to explore the underlying mechanisms of validated circRNAs involved in the tumorigenesis or development of HSCC.

### Interaction between validated circRNAs and potential miRNA targets

Among the predicted miRNA targets of validated circRNAs, several are closely associated with cancer according to published data (Table 3), and these miRNAs are considered promising candidates for future study. We further analyzed their interactions with corresponding circRNAs (Table 4). The analysis included MRE sequences, miRNA seed types, AU-richness near seed sequences, and relative positions of MREs in the linearized sequences of circRNAs. A/U (marked with red bars in Table 4) near MRE facilitates accessibility, whereas G/C (marked with black bars) means low accessibility. The heights of the bars represent the extent of accessibility. Additionally, networks of these cancer-related miRNAs and corresponding circRNAs were built in order to facilitate the molecular mechanism study (Supplementary Figure 2).

**Table 3:** Validated circRNAs and corresponding miRNA targets in cancers

CircRNA ID	miRNA targets	Cancer types	Authors
hsa_circ_0058106	miR-185-3p	Nasopharyngeal carcinoma	Xu J et al.
		Nasopharyngeal carcinoma	Li G et al.
hsa_circ_0058107	miR-185-3p	Nasopharyngeal carcinoma	Xu J et al.
hsa_circ_0024108	miR-185-3p	Nasopharyngeal carcinoma	Li G et al.
	miR-296-3p	Glioblastoma	Bai Y et al.
		Glioblastoma	Lee H et al.
		Prostate cancer	Liu X et al.
		Non-small cell lung cancer	Hu L et al.
		Non-small cell lung cancer	Luo W et al.
	miR-623	Lung adenocarcinoma	Wei S et al.
	miR-670-5p	Hepatocellular carcinoma	Shi C et al
hsa_circ_0036722	miR-1254	Non-small cell lung cancer	Foss KM et al
	miR-145-5p	Hepatocellular carcinoma	Lupini L et al.
		Bladder cancer	Matsushita R et al.
		Prostate cancer	Ozen M et al.
	miR-185-3p	Nasopharyngeal carcinoma	Li G et al.
	miR-671-5p	Breast cancer	Tan X et al.
		Glioblastoma	Barbagallo D et al.

**Table 4:**
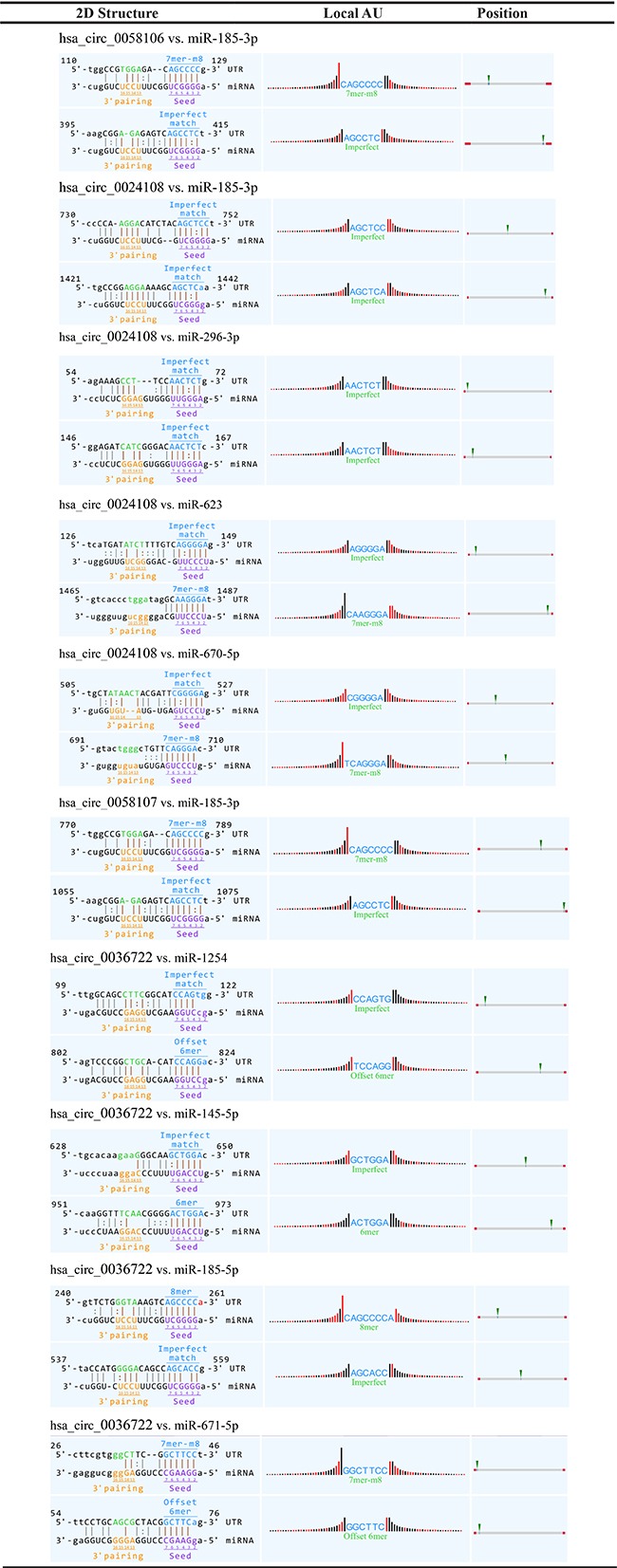
Interaction between validated circRNAs and potential miRNA targets

## DISCUSSION

More recently, circRNAs have become more attention as new diagnostic markers for diseases, including cancers [17, 18, 20–24], while their expression status and roles in HSCC remain to be determined. In the present study, we first utilized circRNA microarray to acquire circRNA expression profiles in HSCC tissues and paired adjacent normal tissues. We identified 2392 significantly dysregulated circRNAs (FC ≥ 2.0, *P* value < 0.05), including 1304 upregulated and 1088 downregulated ones in HSCC tissues. Hierarchical clustering analysis of differentially expressed circRNAs accurately divided detected samples into the tumor and the non-tumor group, further confirming the different expression patterns of circRNAs in HSCC tissues and adjacent normal tissues. These data collectively suggest that circRNAs are involved in the pathogenesis of HSCC.

Genes producing these differentially expressed circRNAs were widely distributed in all chromosomes including sex chromosomes. Intriguingly, upregulated circRNAs in HSCC tissues were predominantly transcribed from chromosome 1 and chromosome 2, while the distribution of downregulated circRNAs was much more even. We believe the distribution status of circRNAs in chromosomes may be related to the biological processes the circRNAs are involved in, as sequences of circRNA largely determine their binding targets.

Validation experiments confirmed the reliability of our microarray data. Moreover, we identified six circRNAs that are definitely dysregulated in HSCC tissues. We speculate that these circRNAs might have a regulatory role in the biological processes of HSCC, which could be verified by loss- and gain-of-function experiments. Additionally, the expression levels of circRNAs in a larger cohort of patient samples should be determined in order to find out whether they can serve as new biomarkers for HSCC diagnosis and whether their expression levels are associated with the clinicopathological characteristics of HSCC patients. Recently, it was demonstrated that circRNAs can be detected in human cell-free saliva [25] and peripheral whole blood [26], suggesting that expression levels of circRNAs in saliva or blood samples of HSCC patients could serve as new, non-invasive biomarkers.

Sponging miRNAs in circRNA-miRNA-mRNA regulatory axes is one of the well-recognized functions of circRNAs such as ciRS-7, CDR1as, and Sry [10]. Using the miRNA target predication software, we preliminarily sought potential miRNA targets of validated circRNAs based on sequence pairing and then constructed networks on the basis of predicted circRNA-miRNA-mRNA regulatory axes. Bioinformatics analysis provided several possible cancer-related pathways for each network, which was helpful for the mechanism study of the validated circRNAs. For example, Zhong, et al discovered the circTCF25-miR-103a-3p/miR-107-CDK6 regulatory pathway in bladder carcinoma through analytical approaches similar to those mentioned above [19].

In the literature, some of these potential miRNA targets have been reported to function as important regulators of proliferation, apoptosis, invasiveness, metastasis, and drug resistance in a variety of cancers, making them valuable for investigation [27–41]. Furthermore, we analyzed their interactions with validated circRNAs. Notably, ectopic expression of miR-185-3p could induce apoptosis and inhibit proliferation in nasopharyngeal carcinoma cells [27]. However, its predicted upstream regulators hsa_circ_0058106, hsa_circ_0058107, and hsa_circ_0024108 are upregulated in HSCC tumor tissues, implying that they might sponge miR-185-3p and then prevent HSCC cells from undergoing apoptosis. Similarly, miR-623 is significantly downregulated in lung adenocarcinoma in comparison with non-tumor tissues and functions as a tumor suppressor [34]. Thus, we speculate that upregulated hsa_circ_0024108 might be an oncogene and act by targeting miR-623. In contrast, miR-671-5p was significantly overexpressed in glioblastoma tissues and its aberrant expression contributed significantly to the migration and proliferation of glioblastoma cells. Considering hsa_circ_0036722 is downregulated in HSCC tissues, we might assume that the deregulated hsa_circ_0036722/miR-671-5p axis might facilitate the invasiveness of HSCC. Additionally, we have requested more HSCC cell lines for *in vitro* experiments for validation in our future studies.

In conclusion, our study for the first time reveals that numerous circRNAs are dysregulated in HSCC. Among the top 10 upregulated/downregulated circRNAs in HSCC, six were validated and deserve further mechanistic study. Bioinformatics analysis predicted several potential circRNA-miRNA-mRNA networks. However, based on these findings, much work is still needed to uncover the underlying molecular mechanisms of circRNAs in HSCC.

## MATERIALS AND METHODS

### Patients and tissue specimens

Tumor tissues and paired adjacent normal tissues were obtained from 36 patients who received surgical treatment for HSCC from June 2015 to April 2016 at Qilu hospital and for whom paired tumor and adjacent normal tissue samples were available. All the patients had a pathological diagnosis of HSCC before surgery, and none was given chemotherapy or radiation therapy prior to the operation. The characteristics of the patients are summarized in Supplementary Table 1. The excised tissue samples were immediately stored in liquid nitrogen overnight and then transferred to −80°C for storage until RNA was extracted. Four of the paired tissue samples were randomly selected for the circRNA microarray analysis. The other 32 pairs of samples were used for validation by qRT-PCR. All subjects provided written informed consent before surgery, and all of the study procedures were approved by the institutional review board of Qilu Hospital.

### RNA extraction

Total RNA was extracted from the tissue samples using the Trizol reagent (Invitrogen, Carlsbad, CA, USA) according to the manufacturer's protocol. The purity and concentration of RNA were determined from OD260/280 readings obtained with the NanoDrop ND-1000 (NanoDrop Thermo, Wilmington, DE, USA). RNA integrity was determined by 1% formaldehyde denaturing gel electrophoresis.

### RNA digestion, amplification, labeling, and hybridization

The extracted total RNA was treated with Rnase R (Epicentre, Madison, WI, USA) to remove linear RNA. The enriched circRNAs were amplified and then transcribed into the fluorescent circRNAs using random primers according to the instructions for the CapitalBio cRNA Amplification and Labeling Kit (CapitalBio, Beijing, China). The amplified circRNA was purified utilizing an RNeasy Mini Kit (Qiagen, Dusseldorf, Germany). The concentration and specific activity of the labeled circRNAs were assessed using the NanoDrop ND-1000. Then the labeled circRNAs were hybridized onto the Agilent human circRNA array (version 1.0; Agilent Technologies, Santa Clara, CA, USA). The slides were rotated at a speed of 20 rpm and a temperature at 42°C in an Agilent hybridization oven overnight, washed, and scanned by the Agilent G2505C Scanner.

### Microarray data analysis

Raw data were extracted from the scanned images by Agilent Feature Extraction software (version 11.0.1.1). GeneSpring software (version 13.0; Agilent Technologies) was used for the microarray data analysis, including data summarization, normalization, and quality control. After normalization of the raw data, low-intensity filtering was performed. The circRNAs with at least 4 of 8 samples flagged as “expression detected” were selected for further analysis. CircRNAs with a fold change in expression ≥ 2.0 (*P* value < 0.05) between the HSCC and normal tissue samples were selected for further analysis. Hierarchical clustering analysis based on expression levels of differentially expressed circRNAs was performed using Java Treeview (Stanford University School of Medicine, Stanford, CA, USA).

### Quantitative reverse transcription and real-time PCR (qRT-PCR)

Extracted RNA was subjected to cDNA synthesis using the SuperScriptTM III Reverse Transcriptase Kit (Invitrogen, Carlsbad, USA), according to the manufacturer's guidelines. Briefly, 3 μg RNA was first mixed with Random N9 primers and dNTP Mix, and then the mixture was put on ice for 2 min followed by incubation in 65°C for 5 min. The reverse transcription system was subsequently prepared, mainly comprising the above mixture, SuperScript III RT and RNase inhibitor. This reaction system underwent successive incubation in water with the temperature of 37°C for 1 min, 50°C for 60 min and then 70°C for 15 min until reverse transcription completed. The detailed components of the two systems used in the reaction were summarized in Supplementary Table 2. qRT-PCR was performed in the ViiA 7 Real-time PCR System (Applied Biosystems, Wilmington, DE, USA) using qPCR SYBR Green master mix (Cloudseq, Shanghai, China) and divergent primer pairs designed for target circRNAs (Sangon, Shanghai, China). The PCR reaction system was presented in Supplementary Table 3. β-actin was used as the internal control. Both target circRNAs and β-actin were amplified as a routine in triplicates with an annealing temperature of 60°C. The relative expression level of each target circRNA was calculated using the 2^−ΔΔCt^ method. Primer sequences were listed in Supplementary Table 4.

### Construction of circRNA-miRNA networks and bioinformatics analysis

Potential miRNA targets of circRNAs were based on miRanda and TargetScan. An interaction analysis of circRNA/miRNA was performed using Arraystar's homemade software, which is based on miRanda and TargetScan. The bioinformatics software Cytoscape was used for the construction of circRNA-miRNA networks. KEGG pathway enrichment analysis of the networks was performed using the KEGG orthology-based annotation system (KOBAS).

### Statistical analysis

Data are presented as means ± standard error (SE). SPSS 17.0 (SPSS Inc., Chicago, IL, USA) was used for the data analysis. The artworks were created by GraphPad Prism 5.0 (GraphPad Software, La Jolla, CA). The Wilcoxon matched pairs test was used for comparing the circRNA expression levels in tumor versus non-tumor tissues. *P* < 0.05 was considered statistically significant.

## SUPPLEMENTARY MATERIALS FIGURES AND TABLES



## Abbreviations

circRNA: circular RNA
HSCC: hypopharyngeal squamous cell carcinoma
qRT-PCR: quantitative real-time polymerase chain reaction
lncRNA: long noncoding RNA
KOBAS: KEGG orthology-based annotation system
