# Supplementary material for: Novel circular RNA expression profiles reflect progression of patients with hypopharyngeal squamous cell carcinoma
Source: Oncotarget. 2017 Apr 27;8(28):45367–79. doi: 10.18632/oncotarget.17488 (PMC5542193; doi:10.18632/oncotarget.17488)
Supplement: Supplementary file 1 [file oncotarget-08-45367-s001.pdf]

Novel circular RNA expression profiles reflect progression of patients with hypopharyngeal squamous cell carcinoma

Supplementary Materials

Supplementary Table 1: Clinical characteristics of patients for microarray and validation by qRT-PCR

For microarray (*n* = 4)						
**Patients**	**Sex**	**Age**	**Smoking**	**Histological Differentiation**	**Clinical Stage**	**Treatment**
1	M	49	Yes	Moderate	II	S + X
2	M	57	Yes	Poor	III	S + X
3	M	65	Yes	Poor	III	S + X
4	M	54	Yes	Poor	III	S + X

For qRT-PCR (*n* = 32)	
Characteristics	No.
Sex	
F	1
M	31
Age (years old)	(Median 53, Range 47-63)
Smoking	
Yes	26
No	6
Histological Differentiation	
Well-Moderate	20
Poor	12
Clinical Stage	
I+II	14
III + IV	18
Treatment	
S + X	32

M: Male; F: Female; S: Surgery; X: radiation.

Supplementary Table 2: Components of the two systems used in the reverse transcription reaction

System 1.

**Components**	**Volume (µl)**
RNA sample (3 µg) and RNase-free water	11.9
0.5 µg/µl Random N9 primers	1
2.5 mM dNTPs Mix	1.6

System 2.

**Components**	**Volume (µl)**
5 × First-Strand Buffer	4
0.1 M DDT	1
RNase inhibitor	0.3
SuperScript III RT	0.2

Supplementary Table 3: System components used for PCR

**Components**	**Volume (µl)**
cDNA	2
2 × SYBR Green master mix	5
10 µM forward primers	0.5
10 µM reverse primers	0.5
RNase-free water	2

Supplementary Table 4: Divergent primers for circRNAs and the internal reference

CircRNA ID	Primer Sequence 5′→3′
hsa_circ_0001189	Forward: GACACAGCTGGTTTCGAAGAG<br>Reverse: ATGATCCTCGTCCCCTTCTT
hsa_circ_0001290	Forward: GGAAAAGAAGCCCAGAAATG<br>Reverse: CGTGCATACTCCTTCACTCG
hsa_circ_0002260	Forward: ATCAAACCTTGGGGACCTCT<br>Reverse: AAAGAGGACCCAACCAAAAA
hsa_circ_0003441	Forward: CCACAGTTCTTGGTGGTGAA<br>Reverse: ATTGATGTCACTGGGGAGGA
hsa_circ_0007646	Forward: AGCTTGGAAATTGGATGCAC<br>Reverse: CAAGACCGCAGACTTCCTGT
hsa_circ_0024108	Forward: GGTGGGGCCCATAAATAAAT<br>Reverse: AGGAGGAGTAGGGGATCGAG
hsa_circ_0058095	Forward: CCATCAAGCCAGATGTCAGA<br>Reverse: CACTGTGACAGCAGGAGCAT
hsa_circ_0058097	Forward: GCCAGACTCCAATCCAGAGA<br>Reverse: ACAAGGCACCATTGGAATTT
hsa_circ_0058104	Forward: TGCACGATGATATGGAGAGC<br>Reverse: TGAATGCCAGTCCTTTAGGG
hsa_circ_0058106	Forward: CTGCAGGTCCAGATCAAACA<br>Reverse: GTTTAAGGCCGCTGATGGTA
hsa_circ_0058115	Forward: GCGAGAGTGCCCCTACTACA<br>Reverse: CAGGAAGGTTGAGTTCTGTGC
hsa_circ_0058121	Forward: CAATGTGGGTCCCTCTGTCT<br>Reverse: ACGGAGTTTGCAGTTTCAGG
hsa_circ_0058143	Forward: GCTTTTGAGCCAACAACTCTG<br>Reverse: ACACACGTGCACCTCATCAT
hsa_circ_0087964	Forward: TCCCCATCCACCTATCTGAG<br>Reverse: CCTGGCCGATGAAGATAGAG
hsa_circ_0088635	Forward: AGGGCAGGCACTAAAACTGA<br>Reverse: GCTGACTTGGGTGACTCTGG
hsa_circ_0002332	Forward: AATCGTGAGCGTTTGTTGAA<br>Reverse: TGCCATGAGTTGATGGAAGA
hsa_circ_0058107	Forward: CTGCAGGTCCAGATCAAACA<br>Reverse: CATGGTGTCTGGACCAATGT
β-Actin	Forward: GTGGCCGAGGACTTTGATTG<br>Reverse: CCTGTAACAACGCATCTCATATT

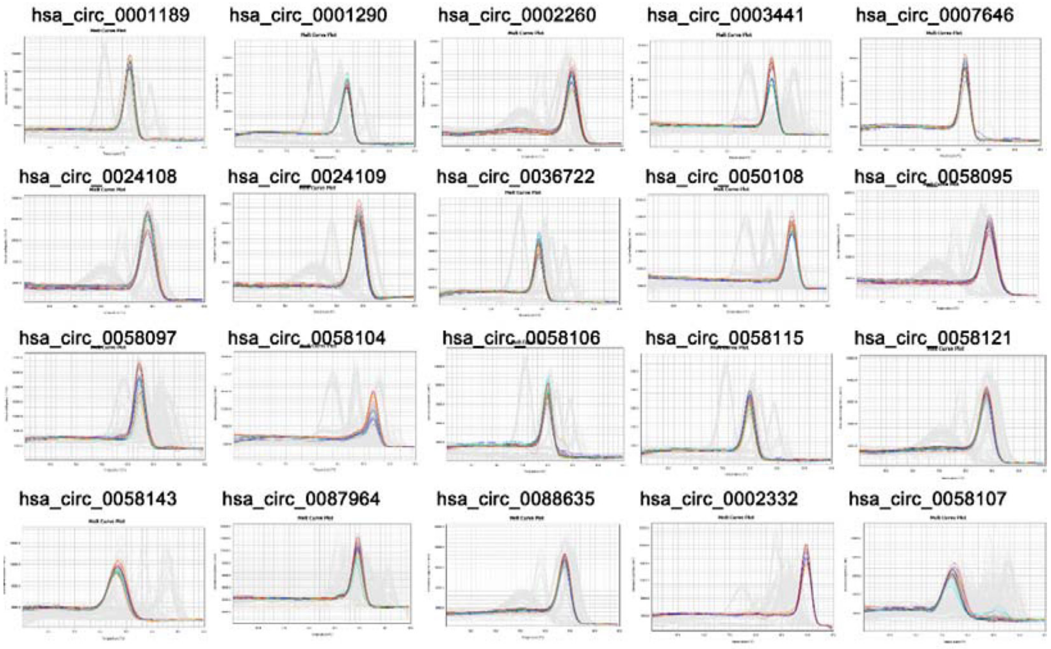

Supplementary Figure 1: Melting curves of top 10 upregulated/downregulated circRNAs in qRT-PCR validation assays.

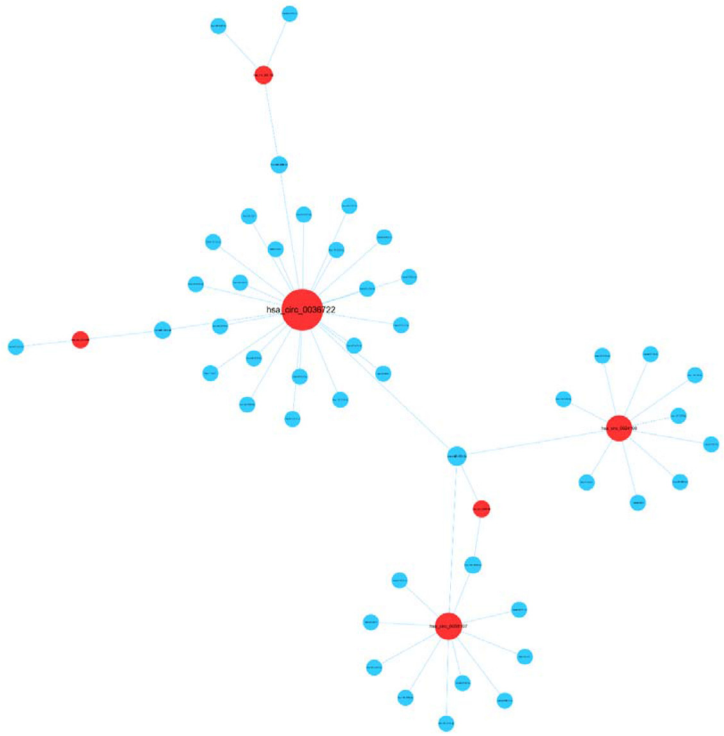

Supplementary Figure 2: Networks composed of validated circRNAs in HSCC tissues and their potential cancer-related miRNA targets.
